# Comparative Evaluation of the Potential Antitumor of *Helleborus purpurascens* in Skin and Breast Cancer

**DOI:** 10.3390/plants11020194

**Published:** 2022-01-12

**Authors:** Ciprian Nicolae Pilut, Aniko Manea, Ioana Macasoi, Amadeus Dobrescu, Doina Georgescu, Roxana Buzatu, Alin Faur, Stefania Dinu, Doina Chioran, Iulia Pinzaru, Monica Hancianu, Cristina Dehelean, Daniel Malița

**Affiliations:** 1 Department of Microbiology, Faculty of Medicine, “Victor Babes” University of Medicine and Pharmacy, Eftimie Murgu Square No. 2, 300041 Timisoara, Romania; pilut.ciprian@umft.ro; 2 Department of Neonatology and Childcare, Faculty of Medicine, “Victor Babes” University of Medicine and Pharmacy, Eftimie Murgu Square No. 2, 300041 Timisoara, Romania; aniko180798@yahoo.com; 3 Departament of Toxicology and Drug Industry, Faculty of Pharmacy, “Victor Babes” University of Medicine and Pharmacy, Eftimie Murgu Square No. 2, 300041 Timisoara, Romania; macasoi.ioana@umft.ro (I.M.); iuliapinzaru@umft.ro (I.P.); cadehelean@umft.ro (C.D.); 4 Research Center for Pharmaco-Toxicological Evaluations, Faculty of Pharmacy, “Victor Babes” University of Medicine and Pharmacy, Eftimie Murgu Square No. 2, 300041 Timisoara, Romania; 5 Department of Surgery II, Faculty of Medicine, “Victor Babes” University of Medicine and Pharmacy, Eftimie Murgu Square No. 2, 300041 Timisoara, Romania; 6 Department of Medical Semiology I, Faculty of Medicine, “Victor Babes” University of Medicine and Pharmacy, Eftimie Murgu Square No. 2, 300041 Timisoara, Romania; 7 Department of Dental Aesthetics, Faculty of Dental Medicine, “Victor Babeş” University of Medicine and Pharmacy, 9 No. Revolutiei Bv., 300041 Timisoara, Romania; drbuzaturoxana@gmail.com; 8 Department of Microscopic Morphology/Histology, Angiogenesis Research Center, Faculty of Medicine, “Victor Babes” University of Medicine and Pharmacy, Eftimie Murgu Square No. 2, 300041 Timisoara, Romania; alin_f32@yahoo.com; 9 Department of Pedodontics, Faculty of Dental Medicine, “Victor Babes” University of Medicine and Pharmacy, 9 Revolutiei 1989 Ave., 300070 Timisoara, Romania; stefania@dr-dinu.com; 10 Department of Dento-Alveolar Surgery, Faculty of Dental Medicine, “Victor Babes” University of Medicine and Pharmacy, 9 Revolutiei 1989 Ave., 300070 Timisoara, Romania; chioran.doina@umft.ro; 11 Department of Pharmacognosy, Faculty of Pharmacy, “Grigore T. Popa” University of Medicine and Pharmacy, 700115 Iasi, Romania; monica.hancianu@gmail.com; 12 Department of Radiology and Medical Imaging, Faculty of Medicine, “Victor Babes” University of Medicine and Pharmacy, Eftimie Murgu Square No. 2, 300041 Timisoara, Romania; malitadani@yahoo.com

**Keywords:** in vitro cytotoxicity, LS-MS, antioxidant activity, flavonoids content, RT-PCR

## Abstract

In the field of oncology, the plant kingdom has an inexhaustible supply of bioactive compounds. Phytochemical compounds isolated from *Helleborus* species have been found to be useful in various chronic diseases. This has brought *Helleborus* to the attention of medical researchers. *H. purpurascens* is a plant characteristic of the Carpathian area, known since ancient times for its beneficial effects. The aim of the study was to evaluate the flavonoids composition of a hydroalcoholic extract of *H. purpurascens*, as well as to assess its antioxidant activity and antitumor potential at the level of two healthy cell lines and four tumor cell lines. In addition, the expression of the genes involved in the apoptotic process (Bcl-2, Bad, and Bax) were evaluated. The results indicated that the extract has a high concentration of flavonoids, such as epicatechin, quercetin, and kaempferol. The extract has an increased antioxidant activity, very similar to that of the standard, ascorbic acid and cytotoxic effects predominantly in the breast cancer cell line, being free of cytotoxic effects in healthy cell lines. Underlying the cytotoxic effect is the induction of the process of apoptosis, which in the present study was highlighted by decreasing the expression of anti-apoptotic genes (Bcl-2) and increasing the expression of pro-apoptotic genes (Bad and Bax). In conclusion, the hydroalcoholic extract of *H. purpurascens* can be considered an important source for future medical applications in cancer therapy.

## 1. Introduction

The threat of cancer continues despite scientific advancements in the medical field. According to data provided by the World Health Organization, cancer was the leading cause of death globally in 2020. The main types of cancer diagnosed, except skin cancer, are breast, lung, and colorectal cancers [1]. Skin cancer, including melanoma and nonmelanoma, has increased sharply in incidence among Caucasians, making them the most common types of neoplasms among this group of people [2]. 

In recent years, remarkable progress has been made in the field of oncology. Thus, the neoplastic process has been more closely studied and understood and, implicitly, the antitumor treatment has shown considerable improvements. Conventional cancer therapy consists of chemotherapy, radiation therapy, and surgery. However, the main disadvantage of classical antineoplastic therapy is the non-selective mode of action which results in the appearance of toxic reactions at the systemic level, as well as the low response rate to treatment [3]. For this reason, special attention has been paid to the plant kingdom which offers an infinite source of compounds with antitumor activity. Phytocompounds have been studied since ancient times, which is why the first antineoplastic drugs have a natural origin. These compounds are characterized by a low toxicological profile and a complex, but selective mechanism of action at the level of the oncogenic signal transduction pathways [4].

Recent research in the field of phytocompounds has shown that *Helleborus* species represent a new treatment opportunity. The genus *Helleborus* is part of the *Ranunculaceae* family and includes over 20 different species with a predominant distribution in Europe and West Asia [5]. Numerous Helleborus plants have been documented for their antitumor, immunomodulating, antioxidant, and cytotoxic potential [6]. In the spontaneous flora of Romania there are mainly two species of *Helleborus*, namely, *H. purpurascens* and *H. odorus*. Although *H. purpurascens* has a high toxic potential, it has been used since ancient times to treat various diseases such as psychiatric and cardiovascular disorders or various pains [7]. Multifaceted therapeutic effects of *H. purpurascens* include muscle relaxation and analgesic properties. However, the exact phytocomponents responsible for these biological activities are not known [7]. Additionally, *H. purpurascens* root extract caused an immunostimulatory effect in vivo by stimulating lymphocytes and neutrophils, [8]. Following the analysis of the composition in phytocompounds contained by *H. purpurascens*, it was determined that this plant has a rich composition of natural compounds with biological effects, such as polyphenols, tannins, and glycosides [9,10]. In light of complex composition of phytocompounds, many studies have investigated the biological effects and mechanisms of action associated with *H. purpurascens* extracts [11]. Regarding the antitumor, antiproliferative, and cytotoxic potential, the studies revealed that this species has an increased potential for use in the cancer therapy and prophylaxis, but the mechanism of action is not yet fully elucidated [10,12]. 

One of the modern directions of antitumor therapy is based on the elimination of cancer cells through the process of apoptosis [13]. Apoptosis is the programmed cell death that in physiological conditions contributes to the maintenance of homeostasis and the elimination of unwanted cells [14]. In terms of cancer, apoptosis plays an important role in preventing the formation of tumors. By losing apoptotic control, cancer cells become more resistant to treatment, and therefore survive longer by becoming more invasive and aggressive. An example of a mechanism that tumor cells use to prevent apoptosis is to increase the expression of the anti-apoptotic protein (Bcl-2) and decrease the expression of the pro-apoptotic protein genes (Bax and Bad). Thus, overexpression of Bcl-2 is common in most cancers, being one of the therapeutic targets of oncology [15].

For this reason, the present study focused primarily on evaluating the composition of a hydroalcoholic extract of *H. purpurascens* (HPex) in terms of the composition of polyphenols. In addition, the antioxidant activity of the extract was determined and the potential cytotoxic effect were assessed on four different cancer cell lines: squamous carcinoma—A431; murine melanoma—B164A5; and breast cancer—MCF-7 and MDA-MB-231. For a complete evaluation of the cytotoxic effect, two healthy cells line were selected: human keratinocyte cell lines (HaCaT) and murine epidermal cells (JB6). Finally, the expression of the main genes involved in the apoptosis process was determined, thus providing a possible explanation for the cytotoxic effect of *H. purpurascens* extract.

## 2. Results

### 2.1. Liquid Chromatography–Mass Spectrometry (LC-MS) Assessment of Extract

The extract obtained was subjected to LC-MS quantification and in Table 1 are presented the polyphenols with a concentration greater than 0.5 µg/g d.m

### 2.2. Antioxidant Activity Evaluation

**Figure 1:**
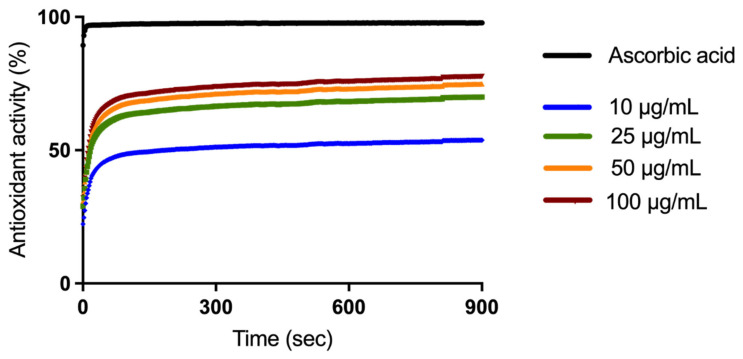
Time-dependent antioxidant activity of the *Helleborus purpurescens* extract.

The (Total Antioxidant Activity) TAOxA of the four different extract concentrations was evaluated by DPPH radical scavenging assay. Ascorbic acid, positive control, exerts a TAOxA of 97.79% as can be observed in Figure 1. The extract samples exhibited an increased TAOxA, in a time-dependent manner over 900 s: 53.83% (10 μg/mL), 69.98% (25 μg/mL), 74.88% (50 μg/mL), and 77.68% (100 μg/mL). 

### 2.3. HPex Exerts a Selective Cytotoxic Effect

Cell viability was determined by the Alamar blue method and expressed as a percentage of viable cells compared to control cells.

**Figure 2:**
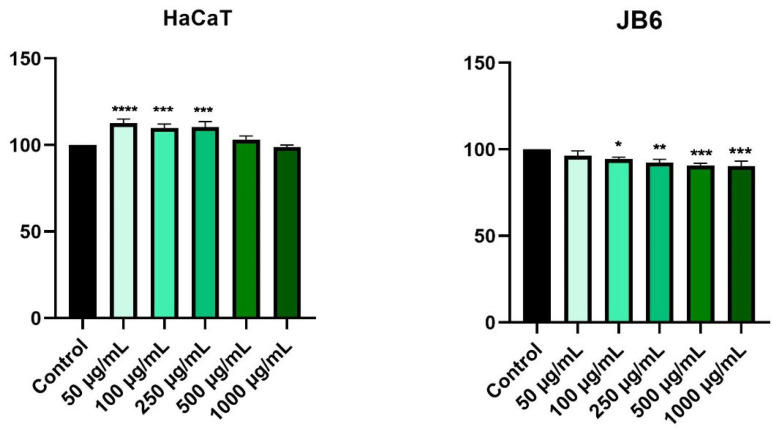
In vitro evaluation of the effect of HPex (50, 100, 250, 500, and 1000 µg/mL) on cell viability on HaCaT and JB6, after 24 h of treatment. The results are presented as cell viability percentage (%) normalized to control (unstimulated) cells and are expressed as mean values ± SD of three independent experiments performed in triplicate. For statistical analysis and comparison between the control and the treated group, one-way ANOVA analysis was applied, followed by Dunnett’s multiple post-test comparisons (* *p* < 0.1, ** *p* <0.01, *** *p* <0.001, and **** *p* <0.0001).

According to Figure 2, stimulation of healthy cells (HaCaT and JB6) with HPex, for a period of 24 h, did not cause significant changes in cell proliferation. Furthermore, HaCaT cells exhibit an increase in viability of approximately 113% for the lowest tested concentration (50 µg/mL), while at the highest tested concentration (1000 µg/mL), the cell viability was similar to that of control cells (approximately 99%). Regarding the JB6 cell line, treatment with HPex resulted in a slight decrease in cell viability. Therefore, the lowest tested concentration held a cell viability value of about 97%, and the highest tested concentration held a cell viability value of about 91% (Figure 2). 

To determine the in vitro cytotoxicity effect, four tumor cell lines were chosen: MCF-7, MDA-MB-231, B164A5 and A43, at which the five concentrations previously tested on healthy cell lines were evaluated.

**Figure 3:**
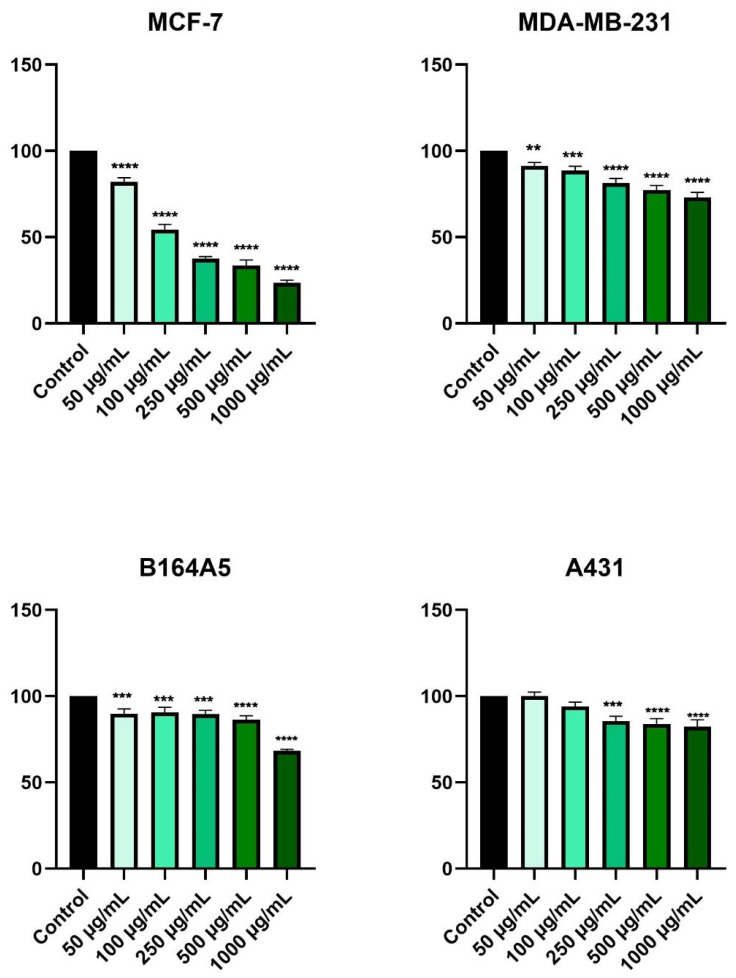
In vitro evaluation of the effect of HPex (50, 100, 250, 500, and 1000 µg/mL) on cell viability on MCF-7, MDA-MB-231, B164A5, and A43, after 24 h of treatment. The results are presented as cell viability percentage (%) normalized to control (unstimulated) cells and are expressed as mean values ± SD of three independent experiments performed in triplicate. For statistical analysis and comparison between the control and the treated group, one-way ANOVA analysis was applied, followed by Dunnett’s multiple post-test comparisons (** *p* < 0.01, *** *p* < 0.001, and **** *p* < 0.0001).

In Figure 3, it is observed that the most affected cells were those of breast adenocarcinoma—MCF-7. In this case, a decrease in cell viability was recorded starting with the lowest concentration tested (50 µg/mL). A concentration of 1000 µg/mL produced the most noticeable cytotoxic effect, where cell viability was about 24% compared to control cells. The second breast cancer cell line, MDA-MB-231, showed a decrease in cell viability in a concentration-dependent manner, but the effect of HPex was not as severe as that of MCF-7 cells. The lowest value of cell viability recorded in MDA-MB-231 cells was observed at a concentration of 1000 µg/mL, approximately 73%.

At the first four concentrations tested, the viability of the murine melanoma cells, B164A5, showed a slight decrease, being around 89%. However, at the highest concentration, cell viability dropped to about 68%. A plateau value of cell viability was also recorded in the case of squamous cell carcinoma, A431, where at the lowest concentration tested cell viability was similar to that of control cells, and at concentrations of 250, 500, and 1000 µg/mL, the values of cell viability were similar, approximately 85%, 84%, and 83%, respectively (Figure 3).

### 2.4. HPex Induces Changes in the Expression of Apoptotic Markers

**Figure 4:**
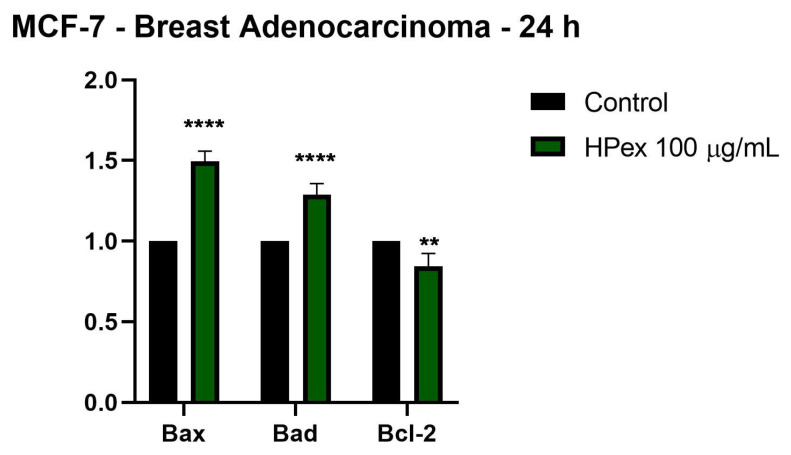
Relative fold change expression of mRNA of pro-apoptotic (Bax and Bad) and anti-apoptotic (Bcl-2) markers in breast adenocarcinoma (MCF-7)—24 h after exposure to HPex 100 µg/mL. mRNA expression levels normalized to 18 S expression, mean values ± SD of three independent experiments presented, one-way ANOVA with Tukey’s post-test used to identify the statistical differences (** *p* < 0.01 and **** *p* < 0.0001).

Following the results obtained in the cell viability test, it was observed that HPex causes a marked decrease in the cell viability of breast cancer cells—MCF-7. In order to provide a more detailed picture of the mode of action of HPex at the level of this cell line, it was decided to determine the expression of the genes involved in the apoptosis process, namely: Bax and Bad—pro-apoptotic genes; Bcl-2—anti-apoptotic gene. Figure 4 shows the effect induced by the sub-cytotoxic concentration of 100 µg/mL HPex. HPex determines upregulation of messenger ribonucleic acid (mRNA) expression for all pro-apoptotic genes analyzed (Bax and Bad), concomitantly with downregulation of mRNA expression of the anti-apoptotic gene (Bcl-2). 

## 3. Discussion

*H. purpurascens* is a widespread plant in Eastern Europe, especially in the Carpathian Mountains. Although the plant has a high toxic potential, it is used in traditional medicine for a wide range of pathologies [16]. Due to its rich content of phytoconstituents, many *Helleborus* species are currently being considered a promising antitumor therapy [17]. Regarding the potential antitumor effect of plants of the *Rannuculaceae* family, including the species of *H. purpurascens*, they were studied for their content rich in active compounds and for antitumor activity [18]. In addition, *H. purpurascens* has been studied for its antitumor effect in vitro, but the biological mechanisms associated with this therapeutic activity have not been fully elucidated [8].

Based on existing scientific considerations, the present study aimed to provide innovative information on the potential antitumor effect of a hydroalcoholic extract of *H. purpurascens*. This therapeutic effect has been studied in conjunction with the evaluation of phytocomposite content, as well as with the evaluation of the beneficial antioxidant effect in antitumor therapy.

To elucidate the cytotoxic effect of HPex, four tumor cell lines were chosen, two of breast cancer (MCF-7 and MDA-MB-231) and two of skin cancer (B164A5 and A431). Additionally, the effects exerted by HPex were studied in two healthy cell lines (HaCaT and JB6), to provide a more accurate picture of the cytotoxic effect. Cell viability was determined using the Alamar blue method, after 24 h of stimulation. Results of the study indicated that HPex has a selective cytotoxic effect, meaning healthy cells are not affected by its use. In contrast, in tumor cells there was a decrease in cell viability depending on the concentration tested. The most visible cytotoxic effects were observed in the breast cancer cell line (MCF-7), where cell viability decreased by up to 24%. The cytotoxic effects were also observed in the other cells studied, but the effect on cell viability was moderate. 

Many *Helleborus* species have been tested for their antitumor potential. In the preliminary study by Lindholm et al. [6], more than 100 plant extracts were tested. Of these, *H. cyclophyllus* extract showed antitumor effects, but the mechanism of action has not been fully elucidated. Another example is represented by *H. caucasicus* which has proven its cytotoxic effects on various tumor cell lines such as lung cancer (A549) or colorectal cancer (DLD-1) [19]. A similar study by Felenda et al. evaluated the antitumor effect of *H. niger* on several tumor cell lines, including melanoma and breast cancer. The results revealed that *H. niger* has a concentration-dependent cytotoxic and antiproliferative effect on all cell lines used [20]. In addition, Schink and colleagues highlighted the cytotoxic effect of *H. niger* extract on the melanoma cell line due to its ability to induce cell apoptosis [21]. Other species such as *H. odorus, H. multifidus*, and *H. hercegovinus* have been tested in vitro on Burkitt’s lymphoma B cells (BJAB). The results revealed that the strongest antiproliferative effect was observed in *H. multifidus* [22]. Regarding the antiproliferative effects of *H. purpurascens* species, previous studies have shown that the alcoholic extract has an antiproliferative effect on the tumor cell line of cervical cancer (HeLa), causing a decrease in the number of mitoses [7]. Voichita et al. also evaluated the effect of two extracts of *H. purpurascens*, an aqueous extract and a hydroalcoholic extract. They determined that both extracts have cytotoxic effects on the HeLa tumor cell line, but the hydroalcoholic extract has a significantly more intense activity than the aqueous extract [10]. Thus, the results obtained in this study support the results presented above and complement them with additional information on the in vitro cytotoxic effect of the hydroalcoholic extract of *H. purpurascens*. 

The B-cell CLL/lymphoma 2 (Bcl-2) family plays a major role in the process of mitochondrial-mediated apoptosis in breast cancer. This family of proteins is divided into two categories: anti-apoptotic members such as Bcl-2 and pro-apoptotic members such as Bax and Bad [23]. A feature of tumor cells is that they avoid the processes of initiating cell death by upregulating members of the Bcl-2 anti-apoptotic family, such as Bcl-2, and decreasing the expression of pro-apoptotic genes, as well as Bax and Bad [24]. Bcl-2 family members also play a major role in resistance to chemotherapy. Due to the fact that they play a crucial role in the regulation of apoptosis, the increased expression of anti-apoptotic genes is correlated with increased resistance to induction of apoptosis and, finally, with resistance to chemotherapy, which underlines the important role played by Bcl- 2 in breast tumors in response to treatment [25]. The present study found that treatment with HPex 100 µg/mL decreased Bcl-2 gene expression, while increased expression of pro-apoptotic Bax and Bad genes. Thus, by decreasing Bcl-2 expression simultaneously with increasing Bax and Bad gene expression, HPex increases the susceptibility of tumor cells to the process of apoptosis. Jesse et al. evaluated the effect of an extract of *H. niger* on the effect on genes involved in apoptosis, mainly the Bcl-2 gene, noting that it induces Bcl-2-dependent cellular apoptosis [26]. Regarding the species *H. cyclophyllus*, Yfanti and collaborators determined that it has pronounced cytotoxic effects in the lung adenocarcinoma cell line, A549. In addition, the group of researchers observed that *H. cyclophyllus* extract induces morphological changes characteristic of the apoptosis process, and in addition, causes a decrease in procaspase-3 levels and cleavage of PARP1 [27].

As a means of better understanding the cytotoxic effect, this study used the LC-MS method to quantitatively determine the phenolic composition of the HP extract. Thus, it was found that the hydroalcoholic extract has a high content of epicatechin, rosmarinic acid, quercetin, and kaempferol, the latter having the highest concentration (approximately 68 µg/g). A wide range of evidence suggested that polyphenolic compounds could be therapeutic in diseases such as cardiovascular disease [28,29,30] and cancer [31,32,33]. Various studies have emphasized polyphenols’ beneficial effects in the treatment of cancer, owing to their antioxidant properties [34,35], pro-apoptotic properties [36,37], anti-proliferative effects [38,39], and interference with the immune system and cell signaling [40,41]. The antitumor effects of polyphenols were first demonstrated by in vitro studies. Such studies have demonstrated the cytotoxic effects of polyphenols in various types of cancer, such as breast cancer [42] and skin cancer [43]. Regarding the extract of *H. purpurascens*, the studies showed a correlation between its polyphenol content and the antitumor effect exerted at the level of different tumor cell lines [8]. The predominant polyphenols in *H. purpurascens* extract, namely epicatechin, rosmarinic acid, quercetin, and kaempferol, have also been studied for their potentially beneficial role in the treatment of cancer. Epicatechin has previously been studied for its effect in the treatment of triple negative breast cancer using a murine model. It has been found to have strong antitumor activity in combination with doxycycline, inhibiting cell proliferation by modulating adenosine monophosphate-activated protein kinase-mediated pathways [44]. Similarly, the effect of epicatechin in the treatment of melanoma was studied, using the melanoma cell line B16F10 and it was observed that it decreases melanin synthesis and inhibits tyrosinase activity, thus participating in the melanogenesis process [45]. An in-depth study of the effect of rosmarinic acid on breast cancer cells, MDA-MB-231, by Messeha et al., elucidated that it had a concentration-dependent cytotoxic and antiproliferative effect, causing increased expression of the Karakiri gene, tumor necrosis factor receptor superfamily 25, and BCL-2 interacting protein to be expressed [46]. Rosmarinic acid also inhibits cell proliferation in the human melanoma cell line A375 through the downregulation of metalloproteinases 17 as well as causing a decrease in melanin [47]. The beneficial role of quercetin in the treatment of breast cancer has been studied in the literature. It seems that this type of flavonoid acts on tumor cells due to its antioxidant activity, causing decreased proliferation and inflammation, stimulating apoptosis and inhibiting angiogenesis and metastasis. Moreover, in the MDA-MB-231 cell line, the cytotoxic effect of quercetin has been linked to increased expression of the *p53* gene, known as a tumor suppressor protein [48]. Additionally, due to the increased expression of the *p53* gene, quercetin has found its utility in the treatment of skin cancer. In addition to this mechanism, quercetin also increases the activity of the tyrosine kinase enzyme, thus causing the antioxidant effect [49]. Kaempferol has an antitumor potential previously discussed in the literature. Its effects have been documented in a variety of cancers, including skin and breast cancer. Among the mechanisms underlying its therapeutic activity are the pro-apoptotic effect, downregulation of epithelial–mesenchymal transition-related markers, and phosphoinositide 3-kinase/protein kinase B signaling pathways [50]. 

However, in addition to its rich flavonoid composition, studies have shown that *H. purpurascens* has many phytoconstants that underlie its antitumor activity. Thus, Franz et al. discussed the composition of aqueous and organic extracts of *H. purpurascens*. Thus, they identified the fact that in terms of aqueous extract, it has a high amount of amino acids, especially asparagine [16]. Similarly, Kumar and co-workers analyzed *H. purpurascens* extract in more detail, providing information on its rich composition in carbohydrates, glycosides, saponins, and tannins [51]. Probably one of the most important cardiotonic glycosides found in the composition of *Helleborus* species is hellebrin, which is found in the largest amount in the species of *H. purpurascens* [17]. Studies on the antitumor effect of natural compounds, highlighted the role played by hellebrin and its deglycosylated form, hellebrigenin, possesses cytotoxic activity on melanoma [52], pancreatic cancer [53], breast cancer [54], and others. The successful use of these compounds in antitumor treatment is due to the inhibitory activity of the Na+/K+-ATPase complex, which prevents the development of cancer cells’ resistance to treatment [52]. In addition to this mechanism of action, hellebrigenin appears to possess antitumor activity based on its ability to induce apoptosis and autophagy of tumor cells [53], but also by inducing an increase in the amount of reactive oxygen species that result in apoptosis [55]. Regarding the steroid-rich composition of *H. caucasicus*, studies have shown that these natural compounds are involved in reducing the viability of tumor cells by inducing apoptosis due to decreased expression of the *GRP78* gene [56]. Similarly, by performing a spectroscopic analysis of the whole plant of *H. niger*, it was determined that due to the composition of bufadienolide and ecdysteroid, the extract has antitumor capacity by inducing apoptosis [57].

**Figure 5:**
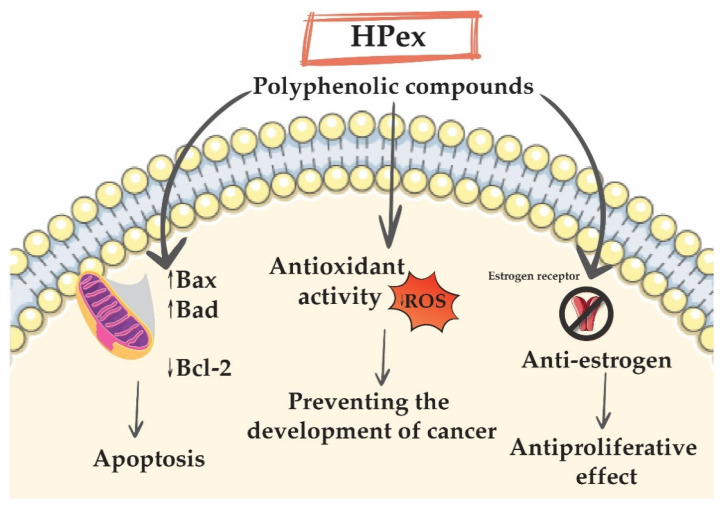
Graphical representation of the possible mechanism of action of Hpex due to its polyphenol content.

As for the MCF-7 breast cell line, it differs from the other MDA-MB-231 breast cancer cell line, mainly due to the fact that MCF-7 is hormone dependent, having positive estrogen and progesterone receptors, while MDA-MB-231 is triple negative. For this reason, MDA-MB-231 does not respond to antiestrogen therapy [58]. Over time, it has been shown that the inclusion of polyphenol-rich foods in the diet of breast cancer patients has been beneficial for the diagnosis and treatment of this type of cancer [59]. Regarding kaempferol, one of the most widespread polyphenolic compounds in the plant kingdom, but also the compound found most in HPex, studies have shown that it has estrogenic activity via ER-mediated pathway causing inhibition of proliferation of MCF-7 cell line [60]. Similarly, quercetin is classified as a phytoestrogen, having an affinity for the type-II estrogen binding site, thereby inhibiting estrogen-mediated cell proliferation and growth [61]. In addition, Meeuwen and colleagues have shown that quercetin is able to inhibit aromatase, thus helping to inhibit the proliferation of MCF-7 breast cancer cells [62]. Accordingly, HPex’s antiestrogen properties might explain the results obtained in the present study, which primarily targeted the MCF-7 breast cancer cell line. Figure 5 shows a possible mechanism of action of Hpex.

In light of the fact that flavonoids are antioxidant compounds [63] and that reactive oxygen species contribute to the development of cancer [64] and other chronic pathologies, such as cardiovascular disease [65,66], diabetes mellitus [67,68], endothelial dysfunction [69,70], or neurological pathologies [71], this study examined the antioxidant activity of the hydroalcoholic extract of *H. purpurascens*. A 2,2-diphenyl-1-picrylhydrazyl (DPPH) radical scavenging assay was applied to evaluate the antioxidant activity of four extract concentrations (10, 25, 50, and 100 µg/mL). The results obtained were related to the antioxidant activity exerted by ascorbic acid and considered 100%. Consequently, the antioxidant activity was higher than 50% in all concentrations, while the most intense antioxidant activity was recorded at the concentration of 100 µg/mL, approximately 78%. Its remarkable antioxidant properties are due to the compounds that were found in *H. purpurascens* extract, which have been associated with the neutralization of oxygen-free species [72]. The antioxidant activity of flavonoids is mainly based on five mechanisms of action, as follows: (i) due to the -OH group in the structure of flavonoids, they interact directly with the reactive part of oxygen radicals; (ii) interaction with the enzyme nitric oxide synthase; (iii) inhibition of xanthine oxidase activity; (iv) preventing the immobilization and adhesion of leukocytes at the endothelial wall and (v) interfering with other key enzymes in the formation of reactive oxygen species such as peroxidase and lipoxygenase [73]. Similarly, the antioxidant activity of two *H. purpurascens* extracts, one aqueous and one alcoholic, were tested. The obtained results showed that the alcoholic extract has a higher antioxidant activity than the aqueous one, with a value of 78% [9]. These results are in agreement with the results obtained in the present study. Similar studies have been performed on other *Helleborus* species. Thus, Öztürk et al. evaluated the flavonoid content and antioxidant activity of an *H. orientalis* extract. They determined that the extract has a significant antioxidant activity, observing an increase in the antioxidant activity and the protective effect against oxidative alterations with increasing concentration of the tested extract [74]. Mohammed and colleagues studied the antioxidant activity of another species of Helleborus, namely *H. vesicarius*. In this study it was determined that this species has an intense antioxidant activity as well as an increased oxidizing potential [75]. Čakar et al. evaluated the antioxidant activity of three species of *Helleborus*, *H. odorus*, *H. multifidus*, and *H. hercegovinus*. In this study, it was determined that the extract made from the leaves has a more intense antioxidant activity than that obtained from the root [22].

## 4. Materials and Methods

### 4.1. Preparation of Extract

The plant material (*Helleborus purpurescens*) used in this study, was harvested from Timis County, Western Romania, and certified at Pharmaceutical Botany Department (voucher herbarium specimen no. HP/S12/2020), Faculty of Pharmacy, “Victor Babes” University of Medicine and Pharmacy. The plant was prepared for extraction according to European Pharmacopoeia (Ph. Eur.) 10th Edition.

To obtain *Helleborus purpurescens* extract (HPex) the maceration method was used. Plant material (5 g), crushed and homogenized, was placed in the flask and 25 mL of ethanol (EtOH) 70% were used to sequentially extraction for seven days. The final extract was filtered through filter paper, the solvent was removed by a rotary evaporator (Heidolph Hei-VAP Advantage Rotary Evaporator package) under vacuum, the pellet being lyophilized and stored in a dark glass tube at 2–8 °C until further analysis.

### 4.2. Liquid Chromatography–Mass Spectrometry Analysis

The hydro-alcoholic hellebore extract, was subjected to a LC-MS analysis, and the following steps were accomplished: (i) extract homogenization (using a WisdVM-10 vortex mixer, Witeg Labortechnik, Wertheim, Baden-Württemberg, Germany) and centrifugation for 120 s at 10,000 rpm (using a ThermoMicro CL17 micro-centrifuge, Thermo Fisher Scientific, Waltham, MA, USA); (ii) LC-MS analysis on a Shimadzu chromatograph (2010 EV, Kyoto, Japan); (iii) chromatographic conditions, extraction time 60 min, room temperature, EC 150/2 NUCLEODUR C18 Gravity SB 150 mm × 2.0 mm × 5 μm column (Macherey-Nagel GmbH & Co. KG, Düren, Germany), flow rate 0.2 mL/min, two mobile phases (mobile phase A—aqueous formic acid; mobile phase B—acidified acetonitrile), wavelengths 280 and 320 nm; (iv) gradient elution—0–20 min 5% B, 20–50 min 40% B, and 50–60 min 95% B; (v) calibration curves between 10 and 50 µg/mL and limit of quantification 0.3 µg/mL.

### 4.3. DPPH Assay

DPPH (2,2-diphenyl-1-picrylhydrazyl) free-radical scavenging assay was selected to evaluate the total antioxidant activity (TAOxA) of hydro-alcoholic HP extract, according to the method described in the literature [76,77]. Briefly, the following steps were realized: (i) a fresh solution of DPPH (10^−4^ M) was prepared in ethanol and 10^3^ µL was added to 3 × 10^3^ µL of hydro-alcoholic HP extracts, at four different concentrations: 10, 25, 50, and 100 μg/mL; (ii) the absorbance was measured continuously for 15 min at 516 nm T70 UV/VIS Spectrophotometer, PG Instruments Ltd., UK); (iii) ascorbic acid was used as positive control, while distilled water as negative control. The final TAOxA was presented as percentage obtained after applying the formula presented in our previous studies [76,77].

### 4.4. Cell Culture

The tumor cell lines selected in the present study were squamous cell carcinoma cell line—A431 (ATCC^®^, Manassas, VA, USA, CRL-1555™); murine melanoma cell line—B164A5 (94042254; ECACC); breast cancer cell lines—MCF-7 (ATCC^®^, Manassas, VA, USA, HTB-22™) and MDA-MB-231 (ATCC^®^, Manassas, VA, USA, CRM-HTB-26™). Healthy cell lines were represented by human keratinocyte cell lines—HaCaT (300493; CLS Cell Lines Service GmbH, Eppelheim, Germania); murine epidermal cells—JB6Cl41-5a (ATCC^®^, Manassas, VA, USA, CRL-2010™). HaCaT, B164A5, A431, and MDA-MB-231 were cultured in the specific culture medium represented by Dulbecco’s Modified Eagle’s Medium high glucose in which were added 10% fetal bovine serum (FBS, Sigma-Aldrich, Bucharest, Romania) and 1% penicillin/streptomycin solution (Pen/Strep 10,000 U/mL; Gibco, Waltham, MA, USA). JB6Cl41-5a and MCF-7 were cultured in Eagle’s Minimum Essential Medium, which was supplemented with 5% fetal bovine serum (FBS, Sigma-Aldrich, Bucharest, Romania) and 0.1% non-essential amino acids for JB6Cl41-5a and 10% fetal bovine serum (FBS, Sigma-Aldrich, Bucharest, Romania) for MCF-7. To prevent microbial contamination, 1% penicillin/streptomycin solution mixture (Pen/Strep 10,000 U/mL; Gibco) was added. During the experiments, the cells were maintained in constant conditions of temperature and humidity (5% CO_2_ and 37 °C). Countess II Automated Cell Counter (Thermo Fisher Scientific, Inc., Waltham, MA, USA), was used to determine the number of cells in the presence of Trypan blue. 

### 4.5. Cell Viability Assessment

In order to determine the cell viability following the treatment with the hydroalcoholic extract of *H. purpurascens* the cells were cultured in 96-well plates in 1 × 10^4^ cells/well. After reaching a confluence of approximately 90%, the culture medium was removed and replaced with 100 μL of fresh medium containing five concentrations of hydroalcoholic extract (50, 100, 250, 500, and 1000 µg/mL). The treated cells were incubated for 24 h. After this interval, cell viability was determined using the Alamar Blue method using the protocol described above [78]. Thus, a volume of 10 µL/well of Alamar Blue was added, and the cells were incubated for three hours. After this interval, the absorbents were determined spectrophotometrically at 570 and 600 nM using the xMark ™ Microplate spectrophotometer (Bio-Rad). Results expressed as percentages of viable cells (%) were calculated using the formula described in one of our previous studies [79].

### 4.6. Gene Expression

Given that, following the cell viability test, the most affected cell line was breast cancer MCF-7, it was decided that the influence of HPex on gene expression should be established by applying the reverse transcription–polymerase chain reaction (RT-PCR) method to this cell line. To determine the expression of the Bax, Bcl-2 (Thermo Fisher Scientific, Inc., Waltham, MA, USA) and Bad (Eurogentec, Seraing, Belgium), the cells were cultured in 6-well plates in a number of 1 × 10^6^ cells/well. After reaching a confluence of approximately 90%, the cells were stimulated with the sub-cytotoxic concentration of HPex (100 µg/mL) for a period of 24 h. After this time, RNA was isolated using Trizol reagent and the Quick-RNA ™ purification kit and its amount was determined using a DS-11 spectrophotometer (DeNovix, Wilmington, DE, USA). Finally, RNA transcription was completed using Maxima® First Strand cDNA Synthesis Kit (Thermo Fisher Scientific, Inc., Waltham, MA, USA), and quantitative real-time PCR analysis was performed using Quant Studio 5 real-time PCR system (Thermo Fisher Scientific, Inc., Waltham, MA, USA) in the presence of Power SYBR-Green PCR Master Mix.

## 5. Conclusions

The present study was designed to determine the chemical composition and biological properties of a hydroalcoholic extract of *H. purpurascens*, a medicinal plant with therapeutic effects applied in traditional medicine since ancient times. LC-MS analysis revealed that the extract contains high levels of flavonoids, especially quercetin, kaempferol, and epicatechin. First of all, the hydroalcoholic extract exerted a strong antioxidant effect, close to that of ascorbic acid, mainly at a concentration of 100 µg/mL. Further, starting from the beneficial role played by antioxidants in antitumor therapy, the hydroalcoholic extract proved its selective cytotoxic effects in the four tumor cell lines used, the proliferation of healthy cells not being affected by stimulation with *H. purpurascens* extract. Finally, the influence of HPex on increasing the expression of pro-apoptotic genes (Bad and Bax) and decreasing the expression of anti-apoptotic genes (Bcl-2) was highlighted in the breast cancer cell line—MCF-7. In conclusion, the extract has an important therapeutic value for the future of oncological therapy, but, nevertheless, additional studies are needed to unravel the mechanism of action and identify the key phytoconstituents which explain these biological effects.

## Figures and Tables

**Table 1:** Individual phenolic compounds quantification by LC-MS in *Helleborus purpurescens* extract.

Standard Phenolic Compound	Rt (min)	Monoisotopic Mass (Da)	m/z	Conc (µg/g d.m.)
Gallic acid	4.74	170.02152329	169	3.075
Proto catechuic acid	11.104	154.02660867	153	0.496
Caffeic acid	20.896	180.04225873	179	3.168
Epicatechin	23.265	290.07903816	289	33.557
p-Coumaric acid	24.310	164.047344113	163	0.879
Ferulic acid	23.457	194.05790880	193	1.893
Rutin	25.925	610.15338487	609	5.546
Rosmarinic acid	29.001	360.08451746	359	21.301
Resveratrol	30.238	228.078644241	227	13.223
Quercetin	31.488	302.04265265	301	46.710
Kaempferol	34.870	286.04773803	285	67.761

## Data Availability

Not applicable.

## Abbreviations

Bcl-2	B-cell CLL/lymphoma 2
DPPH	2,2-diphenyl-1-picrylhydrazyl
EtOH	ethanol
FBS	fetal bovine serum
HPex	hydroalcoholic extract of *H. purpurascens*
LC-MS	Liquid chromatography–mass spectrometry
mRNA	messenger ribonucleic acid
Pen/Strep	penicillin/streptomycin solution
Ph. Eur.	European Pharmacopoeia
RT-PCR	reverse transcription–polymerase chain reaction
TAOxA	Total Antioxidant Activity
